# Development of Specialized Microelectrode Arrays with Local Electroporation Functionality

**DOI:** 10.1007/s10439-023-03268-0

**Published:** 2023-06-16

**Authors:** Andrea Kauth, Anne-Kathrin Mildner, Lena Hegel, Joachim Wegener, Sven Ingebrandt

**Affiliations:** 1 Institute of Materials in Electrical Engineering 1, RWTH Aachen University, Sommerfeldstr. 18-24, 52074 Aachen, Germany; 2 Institute of Analytical Chemistry, Universitaet Regensburg, Universitaetsstr. 31, 93053 Regensburg, Germany; 3 Fraunhofer Research Institution for Microsystems and Solid State Technologies EMFT, Universitaetsstr. 31, 93053 Regensburg, Germany

**Keywords:** Gene electrotransfer, Electropermeabilization, Micro- and nanosystems, Transfection

## Abstract

When a cell or tissue is exposed to a pulsed electric field (100–1000 V/cm), the cellular membrane permeabilizes for biomolecules that cannot pass an intact cellular membrane. During this electropermeabilization (EP), plasmid deoxyribonucleic acid sequences encoding therapeutic or regulatory genes can enter the cell, which is called gene electrotransfer (GET). GET using micro-/nano technology provides higher spatial resolution and operates with lower voltage amplitudes compared to conventional bulk EP. Microelectrode arrays (MEAs), which are usually used for the recording and stimulation of neuronal signals, can be utilized for GET as well. In this study, we developed a specialized MEA for local EP of adherent cells. Our manufacturing process provides a most flexible electrode and substrate material selection. We used electrochemical impedance spectroscopy to characterize the impedance of the MEAs and the impact of an adherent cellular layer. We verified the local EP functionality of the MEAs by loading a fluorophore dye into human embryonic kidney 293T cells. Finally, we demonstrated a GET with a subsequent green fluorescent protein expression by the cells. Our experiments prove that a high spatial resolution of GET can be obtained using MEAs.

**Supplementary Information:**

The online version contains supplementary material available at 10.1007/s10439-023-03268-0.

## Introduction

Transfection defines the transfer of exogenous nucleic acids into a eukaryotic cell, for instance, for gene therapy treating otherwise incurable inherited diseases [8]. Gene electrotransfer (GET) is a non-viral physical transfection method, which offers an immune-friendly and cost-effective alternative to conventional viral transduction [3, 25, 34]. The underlying mechanism of GET is electroporation, or more generally, electropermeabilization (EP). In this procedure, a pulsed electric field with field strengths of typically 100–1000 V/cm between two electrodes is applied to the target cells or tissue [21, 25]. In response, the cellular membrane permeabilizes for biomolecules that could not pass an intact cellular membrane. Thus, for example, plasmid deoxyribonucleic acid (DNA) sequences encoding therapeutic or regulatory genes can enter the cell.

In conventional bulk EP, the cells are suspended in a mixture of plasmid DNA and a special EP buffer. The mixture is placed in a cuvette with a parallel plate electrode arrangement [28]. Since the electric field strength of an ideal parallel plate capacitor scales with the distance of the electrodes (*E* = *U*/*d*), high voltages between the opposing electrodes are required to achieve an electric field strength of about 1 kV/cm as it is required for membrane EP. [4, 28]

Compared to this approach, micro-/nano EP operates with low voltages and provides localized EP [4, 7, 26]. An emerging field in this segment is the in situ EP in which cells are directly adherent to the microelectrodes. Successful in situ EP has also been shown in combination with electric cell-substrate impedance sensing (ECIS) before, during, and after the EP pulse [30, 33]. *In situ* EP approaches have been also described using interdigitated electrodes [1, 10] or specialized microelectrode arrays (MEAs) [15].

MEAs in general enable a high-resolution recording and stimulation of electrophysiological signals from large populations of electrically excitable cells such as neurons [29]. Depending on the application, the electrode surface area is ideally below 1000 µm^2^ [32], while it should not exceed 2000–4000 µm^2^ [9] to provide a decent spatial resolution of the neuronal recordings. Jain and Muthuswamy demonstrated the dual use of MEAs with electrodes sizes of 10000 µm^2^ to combine EP of neurons with electrophysiological recordings [18]. However, they experienced an inhomogeneous electric field distribution across the electrodes. Therefore, they suggested varying the electrode distances and shapes to improve electric field homogeneity [17]. Recently, Duckert et al. have shown successful EP and GET on a high-definition complementary metal oxide semiconductor (CMOS) MEA with over 16.000 individual addressable electrodes with sizes down to subcellular dimensions, providing a very powerful in vitro test setup [11].

Nowadays, clinical GET is intensely investigated in oncology [13] and for vaccinations [14]. In the last two decades, GET was also introduced to the field of ophthalmology [12]. Until now, there are no treatment options available for patients suffering from degenerative retinal diseases such as retinitis pigmentosa (RP) or age-related macular degeneration (AMD). In both diseases, the photoreceptor cell layer degenerates during disease progression [2]. In this respect, subretinal transplantation of genetically modified retinal cells may offer a potential therapeutic intervention. It has been shown that an efficient, stable GET can lead to a continuous expression of the anti-angiogenic and neuroprotective pigment epithelium-derived factor (PEDF) in primary retinal cells [19]. In addition to gene therapy, research is also conducted in the field of retinal prostheses to replace damaged photoreceptor cells. Hereby, MEAs are implanted at different positions of the retina to stimulate the remaining network of the degenerated retina to create a phosphene-based vision [2]. In our research collaboration, we are investigating the possibility of integrating GET into future retinal prostheses to combine local in vivo gene therapy with electrical stimulation of the retina [20].

**Fig. 1:**
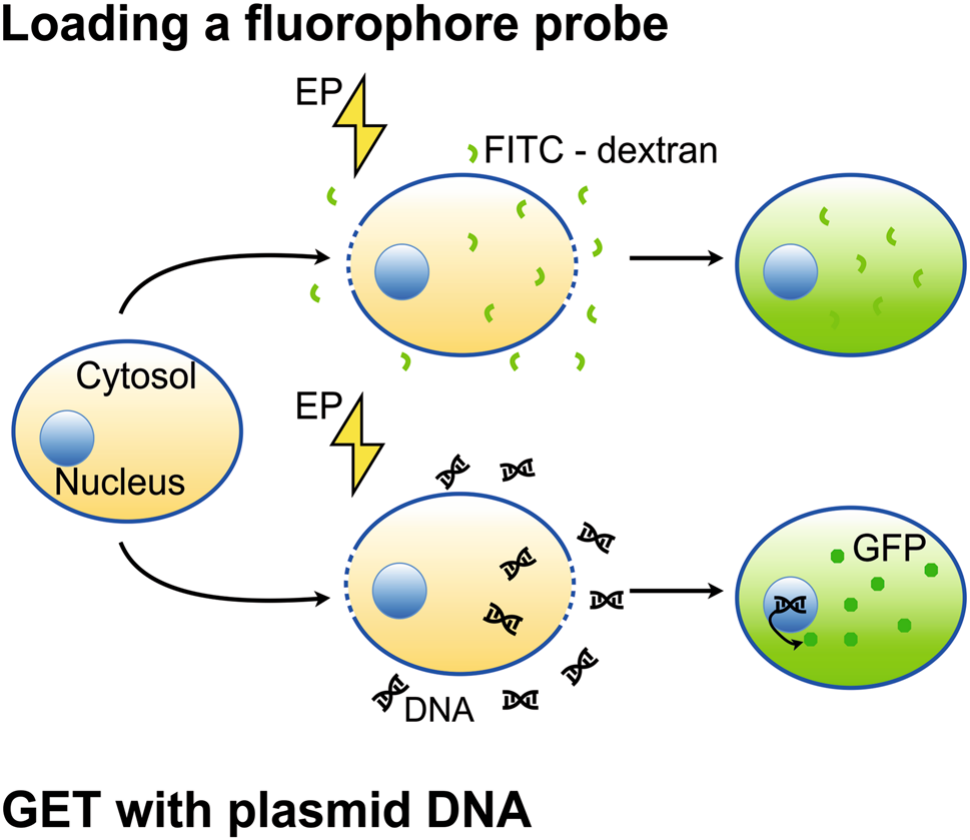
Schematic drawing of the permeabilization of the cellular membrane while applying an electric field. Due to this permeabilization either a fluorophore probe (labelled in green, top part of image) or plasmid DNA (bottom part of image) can enter the cell. If the transfection was successful, the cell can express fluorescent proteins as e.g. GFP (labelled as green circles, bottom part of the image)

We are aiming to develop a specialized MEA that incorporates multiple functionalities such as stimulation, recording, and local EP of adherent cells. Although these MEAs were fabricated specially tailored to in vitro experiments, we additionally designed a cleanroom process to fabricate flexible MEAs for in vivo applications of future retinal implants. The electrode shapes and distances were modified to generate more homogeneous electric fields within the cellular layer. As a proof-of-concept of the local EP functionality, we conducted two sets of experiments: first the EP based-loading of a fluorophore dye and afterward the full GET transfection with subsequent green fluorescent protein (GFP) expression, as it is schematically shown in Fig. 1**.**

## Materials and Methods

### Fabrication and Encapsulation of MEAs

**Fig. 2:**
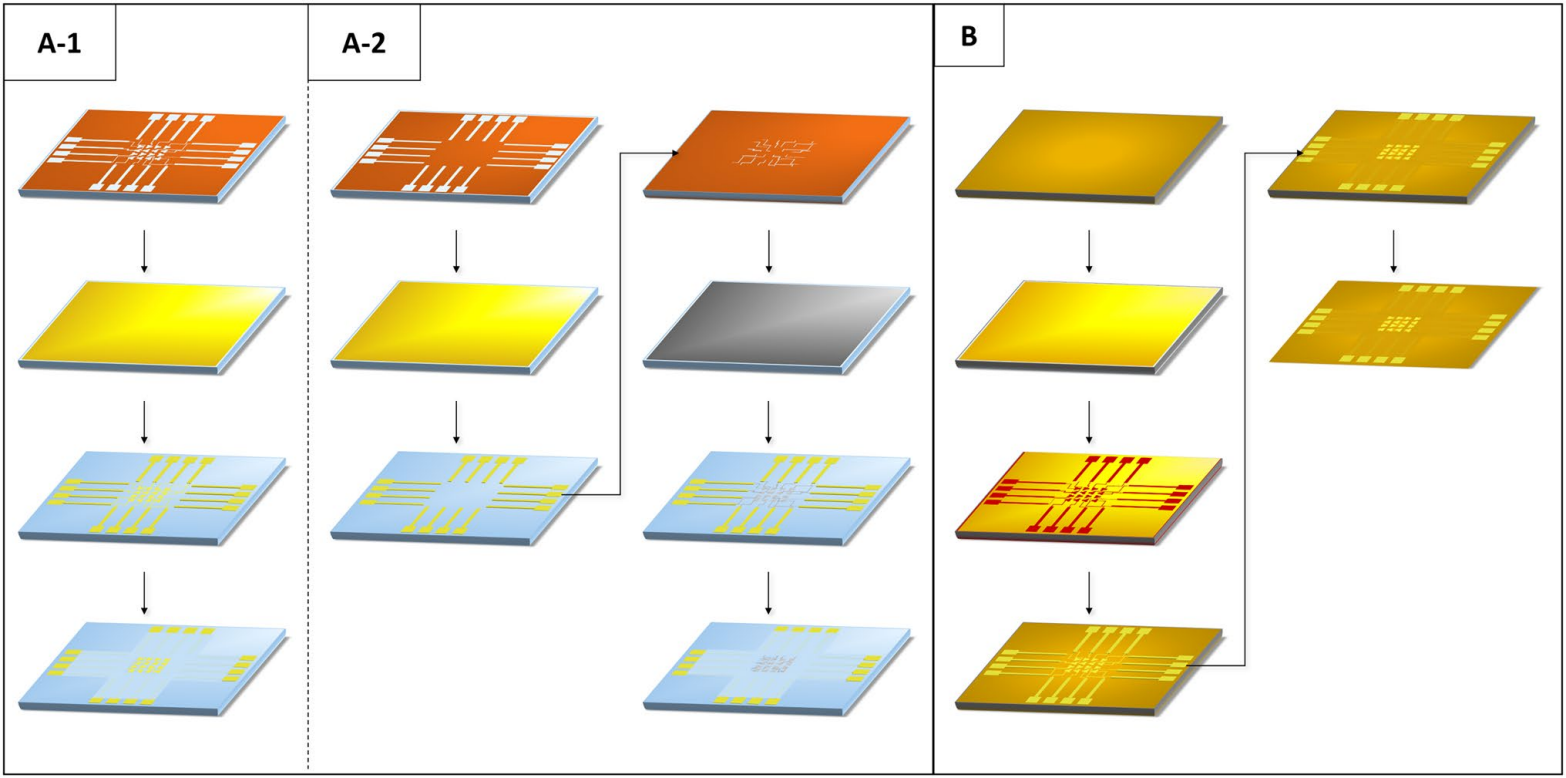
Process steps in cleanroom fabrication: **a-1** After the negative photoresist (red) was patterned, gold deposition followed. By a lift-off in warm dimethyl sulfoxide (80 °C), the metallization was patterned. As a final step, the wafers were coated with Parylene-C as an insulating layer, which was opened at the electrodes and bond pads by dry etching. **a-2** Same process as in A-1 and an additional metallization of another material as electrode material. **b** PI was first patterned and cured on a wafer. This was followed by metal deposition. Then a photoresist was patterned to protect the metal layer and the uncovered areas were etched away. This was followed by a second PI structure as passivation and the last step was to peel the PI off the wafer

Borosilicate glass wafers with 100 mm (4ʺ) diameter were used as substrates for the MEA structures. For a lift-off process (Fig. 2a-1 and a-2), AZ5215E photoresist (MicroChemicals GmbH, Ulm, Germany) was spin-coated and patterned by an image reversal UV photolithography step. Then, 10 nm titanium was deposited as an adhesion layer in a Leybold 700QE evaporation system (Leybold GmbH, Cologne, Germany), followed by an evaporation of 150 nm of gold (Au). Depending on the desired electrode material, some wafers were subsequently sputtered with 200 nm indium-tin-oxide (ITO) in a Nordiko NS 2550 sputter system (Fig. 2a-2). The ITO layer was again patterned by a UV lithography lift-off step. To achieve transparency, the ITO wafers were annealed in a nitrogen atmosphere at 400 °C for 4 h. On all wafers, a 4 µm thin layer of Parylene-C (PDS2010E LabCoter®1, Specialty Coating Systems, Indianapolis, IN, USA) was deposited for feedline insulation. The electrode openings were patterned with a second photolithography step followed by reactive ion etching in oxygen.

**Fig. 3:**
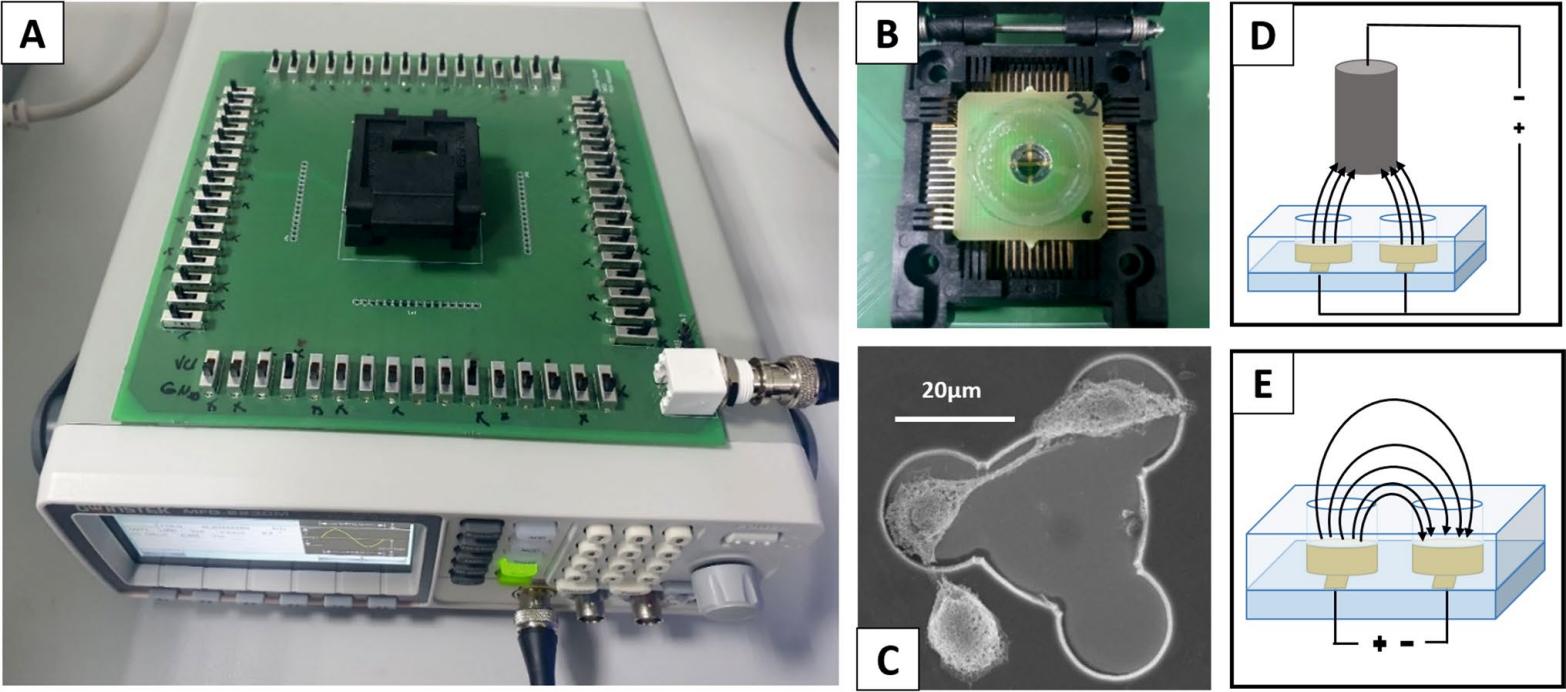
Experimental Setup: **a**:Function generator plugged in via BNC to a PCB that enables to control single electrodes via mechanical switches. **b** IC socket contacting our custom MEA. **c** Scanning electron microscope image of a fixated HEK293 cell on a single electrode of the MEA. **d** Schematic drawing of the electrode arrangement with an external platinum wire as CE, which was used in protocol 1. **e** Schematic drawing of the electrode arrangement without an external CE, which was used in protocol 2

After dicing the wafer into smaller MEA chips, these dies were contacted with silver glue (EPO-TEK® H20E-PFC, Epoxy Technology, Billerica, MA, USA) to printed circuit boards (PCBs). As the PCB material is typically cytotoxic, the PCBs were encapsulated with Polydimethylsiloxane (PDMS) (DOWSIL 96-083, The Dow Chemical Company, Midland, MI, USA). The carrier PCB was designed to be electrically contacted by an integrated circuit (IC) test socket (IC51-0684-390-1, Yamaichi Electronics Co., Ltd., Tokyo, Japan) (Fig. 3b).

In addition, the same MEA structures were fabricated on a polyimide (PI) substrate (PI2611, HD MicroSystems GmbH, Neu-Isenburg, Germany) as a proof-of-concept for future flexible implant devices (Fig. 2b). First, a 5 µm thick PI layer was spin-coated onto a 4ʺ silicon (Si) wafer with a 300 nm silicon-oxide layer, and the solvent was removed at 400 °C in a nitrogen atmosphere. The metallization, consisting of 30 nm chromium (Cr) and 100 nm Au, was deposited by evaporation as on the glass wafers. After the AZ5214E photoresist was structured by standard UV lithography, the Cr-Au layer was wet-chemically etched with aqua regia (nitric acid : hydrochloric acid = 1 : 3) and an acid chromium etch (1 l 264 mM Cerium(IV) ammonium nitrate + 35 ml acetic acid). Subsequently, a second 5 µm PI layer was applied by spin-coating and curing again at 400 °C in a nitrogen atmosphere as we demonstrated before [16]. Finally, the PI layers with the metallization were peeled off the wafers manually.

### Electrochemical Characterizations

Electrochemical impedance spectroscopy (EIS) measurements were performed with a Novocontrol Technologies Alpha-A High Performance Frequency Analyzer (Novocontrol Technologies GmbH & Co. KG, Montabaur, Germany). Impedance spectra between 100 Hz and 10 MHz were recorded at 200 distinct frequencies in total, applying a sinusoidal voltage of 10 mV amplitude. For the MEA electrode characterization, EIS was performed in a three-electrode arrangement using one MEA electrode as a working electrode (WE), a platinum wire as a counter electrode (CE), and a commercial Silver/Silver-Chloride (Ag/AgCl) (Dri-Ref short, World Precision Instruments, Inc., Sarasota, FL, USA) electrode as reference electrode (RE). These measurements were carried out in Dulbecco’s phosphate buffer saline (PBS) (purchased from Sigma-Aldrich Chemie GmbH, Munich, Germany).

To identify the optimum AC frequency for the electroporation pulse, we determined the normalized impedance, which describes the contribution of the cell layer to the total impedance. Hereby, two EIS measurements were conducted: one without the cells and one after 24 h, when the cells completely adhered to the MEA. These measurements were performed in cell culture medium and all 60 electrodes of the MEA were short-circuited to reduce the total impedance of the WE.

### Cell Culture, Electropermeabilization, and Gene Electrotransfer

Human embryonic kidney 293T (HEK293T) cells were cultured in Dulbecco’s modified Eagle’s medium (DMEM) with 4.5 g/l *D*-glucose supplemented with 10 % (v/v) fetal bovine serum (both Thermo Fisher Scientific Inc., Waltham, MA, USA), 100 µg/ml penicillin and 100 µg/ml streptomycin (Sigma-Aldrich Chemie GmbH). Cells were kept in an incubator at 37 °C, 5 % CO_2_ and 95 % relative humidity. Subcultivation was performed twice a week when the cells reached 80–90 % confluence using 0.05 % (w/v) trypsin (Sigma-Aldrich Chemie GmbH) following the standard protocols. For EP experiments, substrates were pre-coated with crosslinked gelatin to promote cell adhesion. The surfaces of the MEA chips were incubated with a solution of 0.5 % (w/v) gelatin (from bovine skin, Sigma-Aldrich Chemie GmbH) in H_2_O for 2 h at room temperature. In order to crosslink the gelatin layer, 2.5 % (w/v) glutardialdehyde (Sigma-Aldrich Chemie GmbH) in H_2_O was added for 10 min. The substrates were rinsed 10 times with water to completely remove the cytotoxic glutaraldehyde. HEK293T cells were seeded onto the MEA chips 24 h prior to EP experiments with a density of 100.000 cells/cm^2^ (Fig. 3c).

#### Loading Cells with a Fluorescent Probe

To study the EP functionality of the MEAs, HEK293T cells were electropermeabilized in the presence of the membrane-impermeable fluorescence dye FITC-dextran (2 MDa, Sigma-Aldrich Chemie GmbH). After the cell layer reached a confluence of 80 % approximately 24 h after inoculation, the culture medium was removed, and the cells were incubated with 2 mg/ml FITC-dextran dissolved in Earle’s balanced salt solution supplemented with calcium and magnesium (EBSS^++^, at Sigma-Aldrich Chemie GmbH). After 30 min incubation at 37 °C and 5 % CO_2_, the MEA chips were placed in the EP setup outside the incubator to apply sinusoidal pulses. The dye was removed 15 min after EP and cells were washed twice with EBSS^++^ to remove all fluorophores that were not introduced into the adherent cells. The dye uptake was verified using an upright confocal laser scanning microscope (CLSM) (Nikon Eclipse 90i, Nikon Corp., Tokyo, Japan) with a 60× water immersion objective.

#### GET

To demonstrate the GET functionality, the pmax-GFP plasmid (from Cell Line Nucleofector^TM^ Kit V, Lonza, Basel, Switzerland), carrying the green fluorescence protein (GFP) gene insert, was introduced into the cells by EP. For this, the cell culture medium was removed 24 h after seeding and the cells were incubated for 30 min with pmax-GFP dissolved in EBSS^++^ before applying the EP pulse. One hour after the pulse, 600 µl cell culture medium was added. To ensure GFP expression, cells were again incubated for 24 h. Subsequently, the cell culture medium was removed, and cells were rinsed twice with EBSS^++^. The protein biosynthesis was documented by CLSM.

As control experiment, all working electrodes were short-circuited to be pulsed simultaneously using a platinum wire as a CE dipping into the buffer from above (Fig. 3d), which is further referred to as *electrode configuration 1*. To verify the MEA design, we also applied pulses to a checkerboard electrode configuration, utilizing only the electrodes of the MEA without an external CE (Fig. 3e) (*electrode configuration 2*). An HP 33120A arbitrary waveform generator (Agilent Technologies Inc., Santa Clara, CA, USA) provided the voltage pulse. The pulses were delivered to the MEA via a customized PCB that enabled individual electrodes to be addressed in any configuration.

To estimate transfection efficiency, we applied a cell fixation and nucleus-staining protocol to four MEAs pulsed in *electrode configuration 2*. All chemicals for this protocol were purchased from Sigma-Aldrich Chemie GmbH. First, we removed the cell culture medium after 24 h of incubation after the GET experiments and rinsed the samples once with EBSS^++^. Subsequently, we added 30 µl of 4 % (w/v) paraformaldehyde solution (PFA) to the cells and incubated them for 10 min at room temperature. We then removed the PFA solution and rinsed the MEAs three times with PBS. After the last rinsing step, we added 30 µl DAPI (DNA-binding dye 4′,6-diamidino-2-phenylindole, 10 ng/ml) solution and waited another 5 min until we again rinsed the MEAs three times with PBS before imaging the MEAs at the CLSM. Cell counting was performed in ImageJ [27] using a *RenyiEntropy*-based thresholding, *watershed algorithm* and the function *analyze particles* with a particle size > 20 µm^2^*.* We performed this protocol on both the blue and the green channel, separately. Before applying the protocol, we cropped the CLSM images to the MEA electrode area, which comprises 627 × 680 µm. Single steps of the cell-counting algorithm are depicted in Supplemental Fig. S1.

## Results

### Development of MEAs

**Fig. 4:**
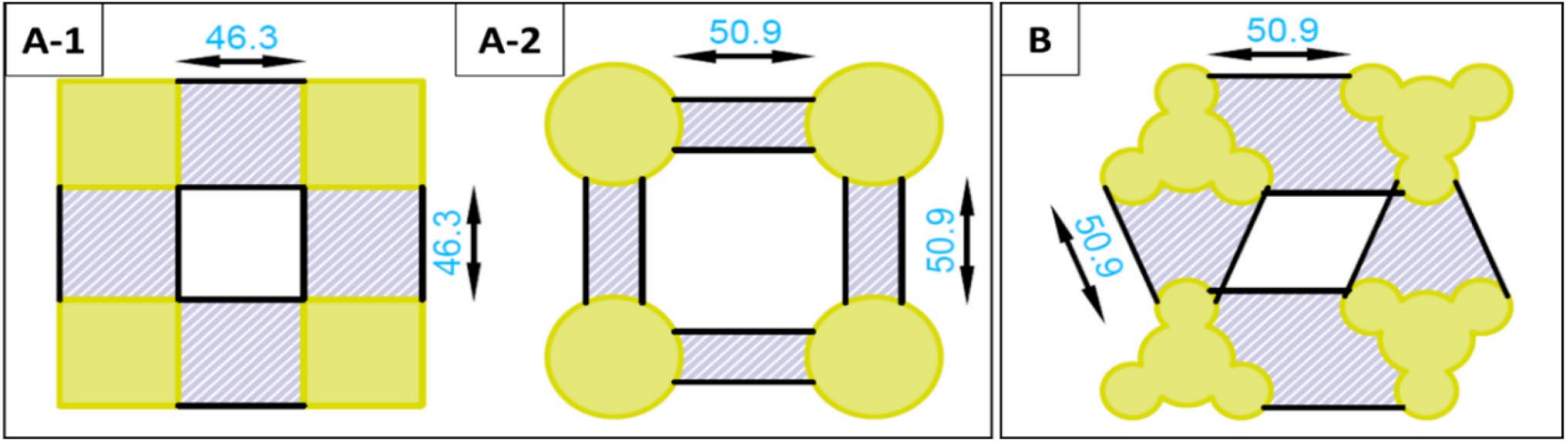
**a-1** and **a-2** show standard MEA electrode geometries**:** The shaded area indicates a region in which the distance between the electrodes varies by a maximum of 10 %. In **a-1** this corresponds to 8084.5 µm^2^ and in **a-2** to 3983.8 µm^2^. Our optimized electrode design is depicted in **b**. Here the shaded area corresponds to 8007.4 µm^2^. Dimensions are documented in µm

To optimize the uniformity of the electric field distribution, we adjusted the electrode geometry. In a former project (unpublished data by F. Waschkowski, A. Garcia Moreno & W. Mokwa, 2016), different electrode shapes were developed to optimize the uniformity of the electric field distribution. The most promising design combined circular shapes in a triangular arrangement (Fig. 4b), in which the intersections were aligned to have equal spacing between all points of maximum proximity.

**Fig. 5:**
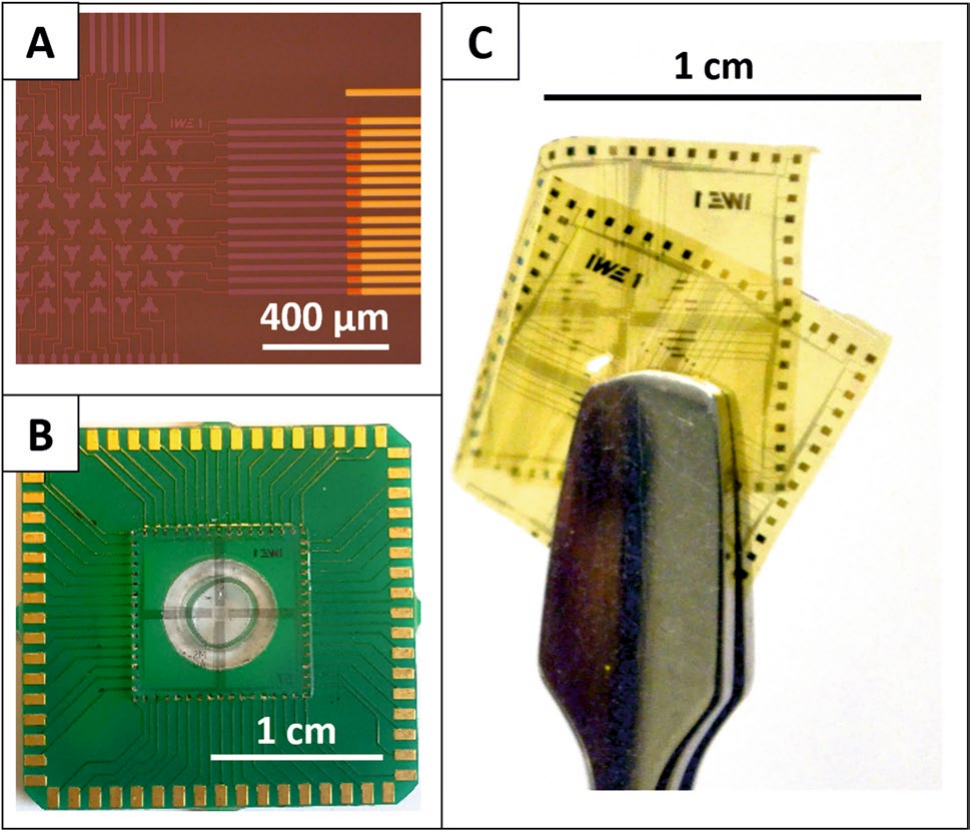
**a** Fabricated MEA with ITO as electrode and inner feedline material. **b** MEA glass-chip mounted on a PCB carrier. **c** Results of the flexible fabrication process

We slightly adjusted the former designs to enable the fabrication of the electrodes and the inner feedlines in one metallization step with a minimum spacing of 5 µm to ensure a reasonable yield in the lift-off lithography process. A single electrode now covers a surface of 1910 µm^2^. With the new electrode design, we avoided sharp edges, which usually cause high electric field strengths as for instance with square electrodes (Fig. 4a-1). With our design, we kept almost the same area in which the distance between two electrodes varies by a maximum of 10 % to ensure a more homogeneous electric field compared to round electrodes Fig. 4a-2). We decided on processing recessed electrodes since it has been shown that these contribute to a more uniform distributed current density [31]. Instead of sizing the glass chips to fit directly into the IC socket, we developed a high-throughput fabrication process. Here, we divided the area of a 4-inch wafer into smaller chips measuring 1.1 × 1.1 cm and mounted them on our PCB carrier, which measures 2.4 × 2.4 cm, using flip-chip assembly (Fig. 5b). In this way, we were able to produce four times as many MEA dies, resulting in a total of 40 chips per wafer.

To achieve maximum flexibility in material selection, we designed different mask sets that allow us to use either the same material for the electrodes, inner and outer feedlines, or combinations of different materials, as e.g. a combination from Au and ITO (Fig. 5a). ITO can be fabricated transparently but it is less conductive than Au. Therefore, combinations of both materials are advantageous.

### Characterization of MEAs and the Cellular Layer

EIS measurements of a single gold electrode revealed an absolute impedance of 10 kΩ at 1 kHz, which is also suitable for neuronal cell stimulation and recording. ITO electrodes revealed an impedance of around 1 MΩ at this frequency, which is the highest acceptable value for neuronal electrodes [9]. Since there are many reports of EP with Au electrodes [5, 10, 15, 18, 30], we performed all characterizations and EP experiments with Au electrodes for comparability.

**Fig. 6:**
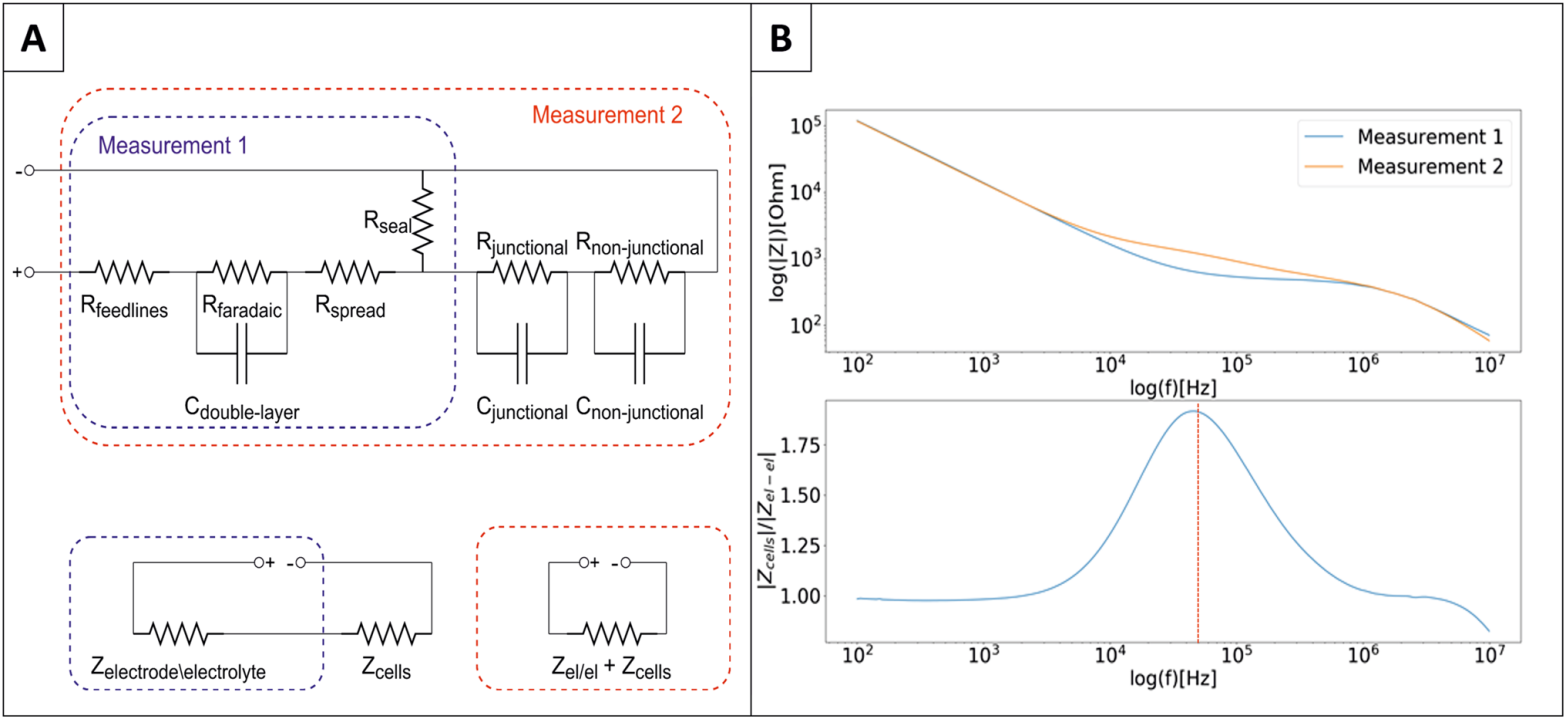
**a** Top: Full EEC describing the impedance of the electrode-electrolyte interface and the impedance contribution of the cellular layer to it (modified from [24, 29]). “Non-junctional” describes the parts of the cellular membrane that face only the electrolyte whereas” junctional” describes the adherent part of the cellular membrane. *R*_seal_ comprises the resistance of the electrolyte (in measurement 2 the electrolyte contribution in the cleft between cells and electrode) to ground. The blue and the red square identify which conditions we measured separately. Bottom: Simplifications of the full EEC in order to identify the AC frequency that shows the biggest impact of the cell layer to the overall impedance. At this frequency, EP pulses deliver a maximum fraction of the totally applied voltage to the cell layer and not to the electrode interface or the bulk. **b** EIS spectrum of MEA with (measurement 2) and without cells (measurement 1) and the resulting normalized impedance. The red dashed line indicates 40 kHz

Several models exist to accurately describe the electrical behavior of an electrode-electrolyte interface and the impedance contribution of the cell layer on it [24, 29]. However, to determine a suitable frequency range for the EP pulses, the complexity of these equivalent electrical circuits can be reduced to a series connection of two complex impedances, one summarizing all contributions of the electrode-electrolyte interface and the other one the contribution of the cell layer (Fig. 6a).

By calculating the normalized impedance (|*Z*_cell_covered_(*f*)|/|*Z*_cell_free_ (*f*)|), the frequency with the strongest cell contribution to the total impedance can be identified as the maximum of a bell-shaped curve. In our experimental conditions, a peak at around 40 kHz (compare Fig. 6b) occurred. At this peak, the voltage drop across the adherent cells is largest and with this, the electric field strength at the cellular membrane is at its maximum. A similar frequency range was already observed previously on ECIS electrodes. [30]

### EP & Transfection

**Fig. 7:**
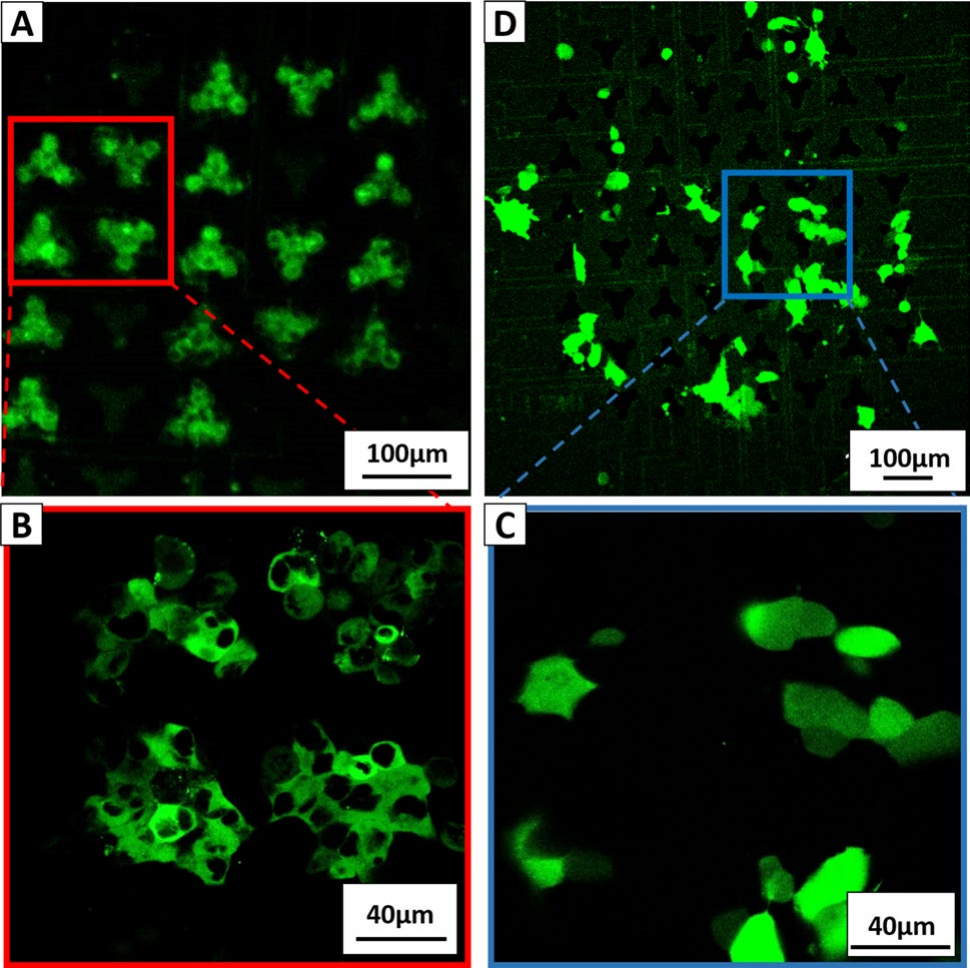
CLSM images showing the results of the preliminary experiments. **a** FITC dextran captured in the cytoplasm of HEK293T cells shown with 100× magnification. **b** Same scenery as A1 with higher magnification (600×). Here it can be seen that the FITC dextran did not enter the nucleus. **c** 100× magnification of transfected cells showing a GFP expression. **d** 600× magnification reveals that the GFP is small enough to enter the nucleus

To test the EP functionality with our MEAs in general, we first cultured HEK293T cells on the MEAs and incubated the attached cells in EBSS^++^ supplemented by FITC-dextran (2 mg/ml). Considering the electrochemical characterization of the MEAs, we applied sinusoidal voltages of 5 V at 40 kHz for 200 ms in *electrode configuration 1*. The amplitude and pulse duration was based on previous experiments [30, 33]. After applying the pulses, we rinsed the MEAs twice with pre-warmed EBSS^++^ and imaged them immediately (Fig. 7a). With this experimental setup, we demonstrated very precise and local EP within the electrode areas of adherent HEK293T cells. The large FITC-dextran molecules were able to enter the cytoplasm but not the nucleus (Fig. 7b).

In the next step, we performed GET experiments with the pMAX GFP DNA plasmid. For this, the plasmid was diluted with EBSS^++^ to a concentration of 500 ng/µl. The adherent cells on the MEAs were incubated with this plasmid solution. After applying the same pulses as before, the MEAs were incubated, and cell culture medium was added. After 24 h, the CLSM showed successful GET of HEK293T cells (Fig. 7c). The GFP produced was small enough to diffuse even through the pores of the nuclear membrane, which is why the entire cell, and not just the cytoplasm, fluoresces green (Fig. 7d).

**Fig. 8:**
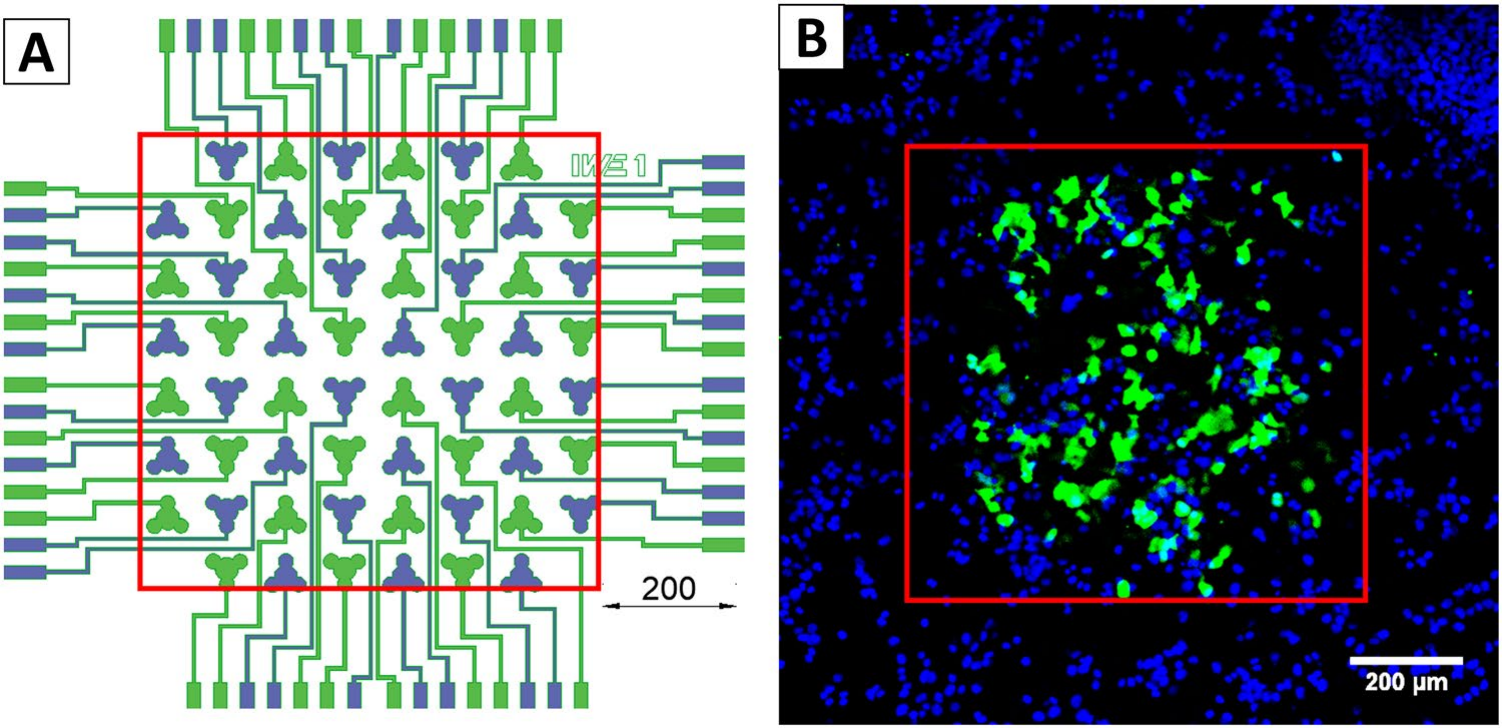
**a** Electrode arrangement used in protocol 2. During pulses, all purple electrodes where at the same time-dependent sinus potential and all green electrodes were grounded. Dimension is depicted in µm. **b**: Image captured at 100× magnification by CLSM. Cells were expressing GFP after 24 h in a local area around the electrodes (red square). DAPI expression reveals all nuclei of the cultured cells

As in future retina implants we aim to integrate this local EP functionality, using an external platinum wire as CE will not be feasible. Towards this aim, we applied the pulses with a bi-polar checkerboard arrangement of electrodes (Fig. 8a) avoiding a macroscopic CE. For this purpose, 30 electrodes each were short-circuited to form two separate electrode clusters (*electrode configuration 2*). The sinusoidal voltage signal was applied to one cluster as WE, while the other cluster served as a CE and was grounded.

CLSM imaging proofed the high spatial resolution of our MEA-based GET, since clearly only cells inside the array were transfected (Fig. 8b). Transfected cells were identified on electrodes from both clusters indicating a symmetric effect by this method. Further CLSM images of different MEAs can be found in Supplemental Fig. 2.

Table 1 represents the results of the cell-counting algorithm in ImageJ. In general, the calculation of transfection efficiencies is difficult, since cells keep on proliferating during culture and eventually detach during experiments and cultivation. Since the plasmid vector we used in our experiments only leads to a transient transfection and will not be replicated, the number of GFP expressing cells will stay constant, whereas the total number of cells will further increase. After EP, the expression of GFP takes several hours and therefore we imaged the cultures after 24 h. When comparing the number of counted nuclei, one notices that it differs and fluctuates, significantly. To estimate the transfection efficiency, we therefore assume the HEK293T cells double every 20 h [22] and with this calculate the transfection efficiency as $\mathrm{efficiency}=\frac{\# c\mathrm{ell GFP}}{\# \mathrm{cell nuclei}}\bullet 2$. This calculation results in transfection efficiencies between 20 and 70 %.

**Table 1:** Results from the cell-counting algorithm in ImageJ

Name	MEA 1	MEA 2	MEA 3	MEA 4
Figure	Supplemental Fig. S2a	Supplemental Fig. S2b	Supplemental Fig. S2c	Fig. 8b
# Cell nuclei in cropped image	276	342	604	410
# Cells with GFP expression	94	51	63	145
Transfection efficiency	68.1 %	29.8 %	20.9 %	70.7 %

The first row lists the images. The second row comprises the number of cell nuclei derived from the blue channel and the third row shows the amount of GFP expressing cells derived from the green channel. The last row comprehends the resulting transfection efficiencies

## Discussion

Local EP via MEAs combines several advantages at once, such as precise spatial control of the target cells or tissues that get electropermeabilized and drastic reduction of the required voltage amplitudes. Smaller electrodes offer a higher resolution on the one hand, but, on the other hand, have higher impedance, which reduces the voltage drop across the cells/tissues. Identifying suitable electrode geometries is therefore essential.

In this paper, the design and fabrication of special MEAs incorporating local EP functionality were presented. First, new electrode shapes were developed to combine the advantages of circular (moderate electric field strength) and square electrodes (proximity of electrodes) and to compensate for their disadvantages, namely a high degree of non-uniformity and excessively high field strengths at the edges. The final geometry of the electrodes also considered the minimum dimensions that ensure a reproducible clean room manufacturing process. We have demonstrated a high degree of flexibility in the cleanroom process and introduced our multi-material MEAs as well as our MEAs on flexible substrates that could be used for in vivo implants in future.

Electrochemical characterizations have shown that these MEAs could be used for neural recording in principle. However, this has yet to be verified by actual neural recording and stimulation experiments. Although the 1 kHz impedance is in a meaningful range for neuronal recording, electrode impedance could be further reduced by coating the electrodes with iridium oxide or Poly(3,4-ethylene-dioxythiophene)-poly(styrenesulfonate) (PEDOT : PSS) [6, 23]. In addition, these materials have a higher charge injection capacity [6], injecting more charges into the cells or tissue at the same voltage compared to gold or platinum, which is beneficial for both neuronal stimulation and EP.

In control experiments, we demonstrated the capability of local EP using our specialized MEAs in *electrode configuration 1*. In particular, fluorophore uptake showed a high spatial resolution of EP (Fig. 7a). Some electrodes showed no fluorescent cells, due to various reasons, such as the electrode being damaged during fabrication or encapsulation, or fewer cells being attached during EP or detached during rinsing with EBSS^++^. Interestingly, the images of the GET experiments also showed cells expressing GFP that were not in direct contact with an electrode but were located between electrodes. However, this could be explained by migrating cells, as we had to wait 24 h for expression to be visible. We conducted successful GET experiments in both electrode configurations with a meaningful transfection efficiency of up to 70 % in *electrode configuration 2*. We are optimistic that further tuning of amplitude, pulse duration, and the number of pulses will further improve the transfection efficiency. However, the transfection efficiencies in our experiments were difficult to estimate, due to many factors such as ongoing cell proliferation, uneven density of seeded cells from chip to chip, detachment during DAPI staining and fluorophore bleaching (Supplemental Fig. S2c and d), and possible errors during automated cell counting.

Although other groups already presented GET on MEAs with higher efficiency [11], to the best of our knowledge, we are the first group to implement a simple and highly adaptable clean room process to fabricate specialized multifunctional MEAs with local EP functionality. Since our fabrication process allows the systematic screening of different electrode materials and also integration on flexible substrates, our method forms the basis for further development of retina implants with the possibility of efficient, local in vivo gene transfer.

### Supplementary Information

Below is the link to the electronic supplementary material.

Supplementary file1 (DOCX 3601 kb)
