# Supplementary material for: Development of Specialized Microelectrode Arrays with Local Electroporation Functionality
Source: Ann Biomed Eng. 2023 Jun 16;52(1):12–21. doi: 10.1007/s10439-023-03268-0 (PMC10761456; doi:10.1007/s10439-023-03268-0)
Supplement: Supplementary file 1 — Supplementary file1 (DOCX 3601 kb) [file 10439_2023_3268_MOESM1_ESM.docx]

Electronic Supplementary Material

**Supplemental Figure 1:**


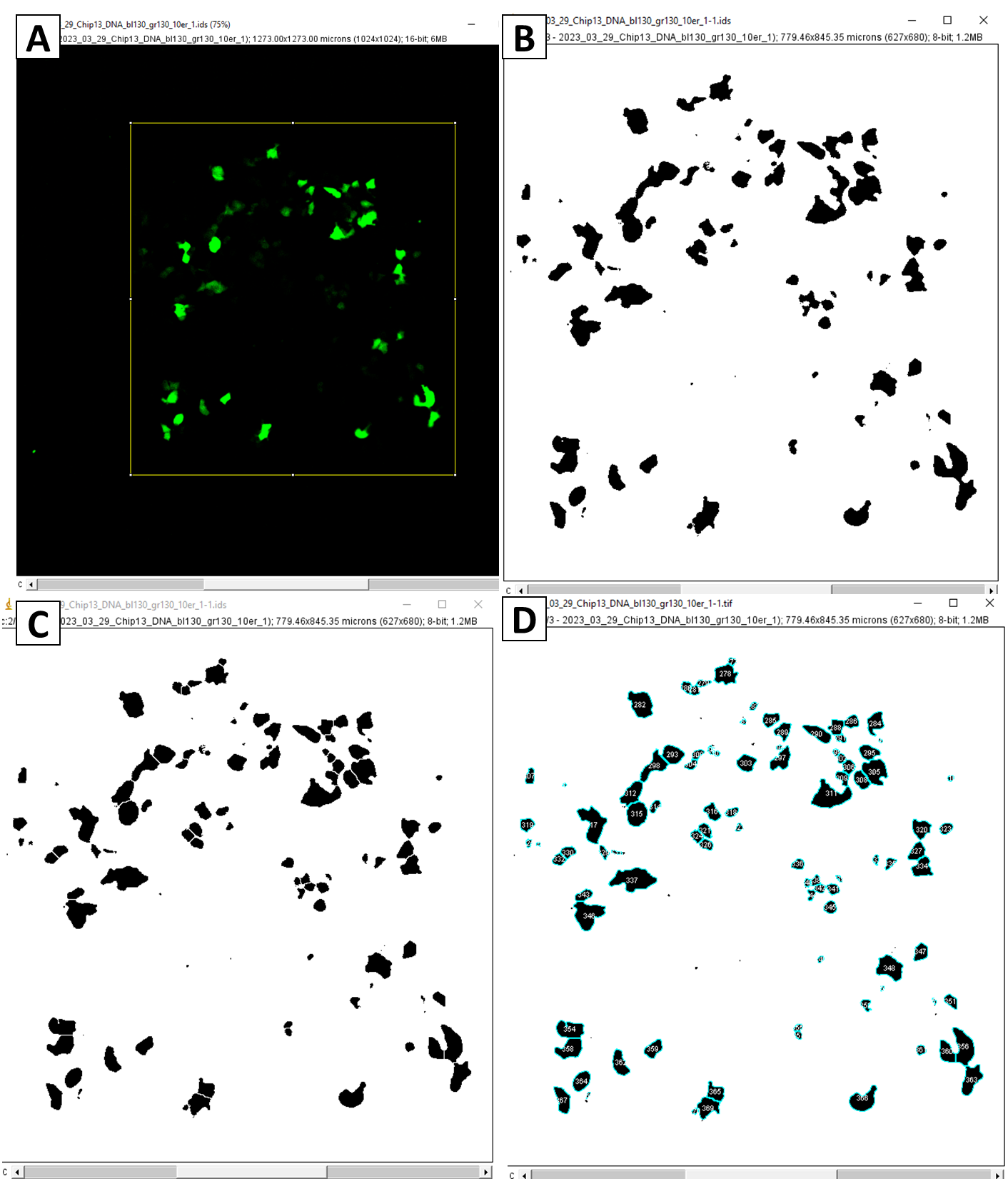


**Supplemental Figure 1:** Cell-counting algorithm. **A**: Original image which is cropped to the size of the MEA electrodes (627 x 680µm). **B:** Image segmentation after thresholding using the ‘RenyiEntropy’ algorithm. **C:** Dividing of larger cell clusters with the ‘Watershed’ algorithm. **D:** Count cells using the ‘Analyze particles’ function.

**Supplemental Figure 2:**


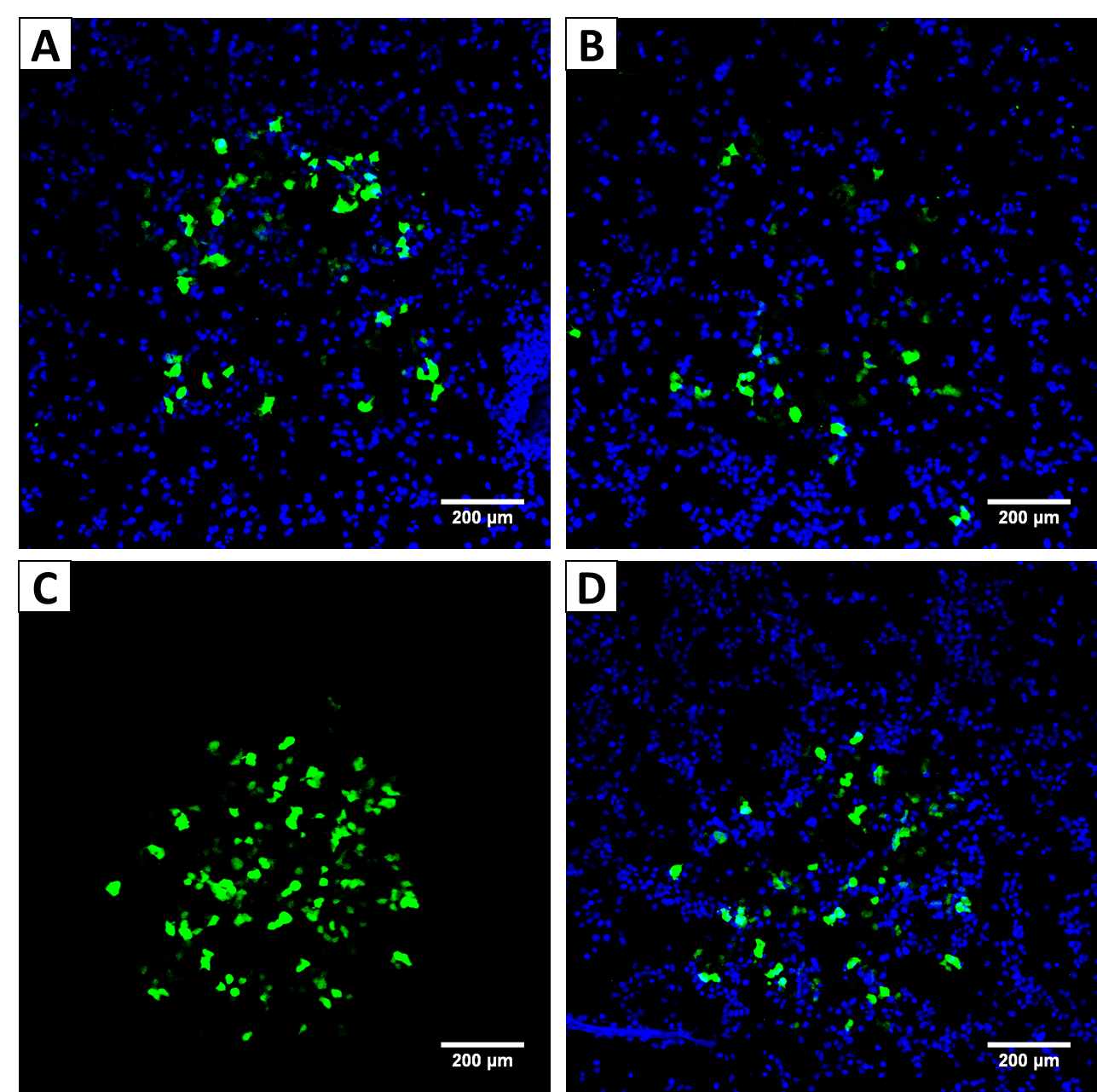


**Supplemental Figure 2:** CLSM images at 100x magnification showing GFP expression after successful GET. **A & B**: To different MEAs with different transfection efficiencies while applying the same pulse parameters. **C:** MEA that was imaged by CLSM before DAPI staining. **D**: Same MEA as **C** after DAPI staining, which is now showing a reduced GFP expression.
